# Temporal Microbial Community Dynamics Within a Unique Acid Saline Lake

**DOI:** 10.3389/fmicb.2021.649594

**Published:** 2021-06-24

**Authors:** Noor-Ul-Huda Ghori, Michael J. Wise, Andrew S. Whiteley

**Affiliations:** ^1^School of Agriculture and Environment, The University of Western Australia, Perth, WA, Australia; ^2^The Marshall Centre of Infectious Diseases, School of Biological Sciences, The University of Western Australia, Perth, WA, Australia; ^3^Department of Computer Science and Engineering, The University of Western Australia, Perth, WA, Australia; ^4^Centre for Environment and Life Sciences, CSIRO Land Water, Perth, WA, Australia

**Keywords:** poly-extremophilic, acid saline lake, taxogenomics, amplicon sequencing, Haloacidophiles

## Abstract

Lake Magic is an extremely acidic, hypersaline lake found in Western Australia, with the highest concentrations of aluminum and silica in the world. Previous studies of Lake Magic diversity have revealed that the lake hosts acid- and halotolerant bacterial and fungal species. However, they have not canvassed microbial population dynamics across flooding, evapo-concentration and desiccation stages. In this study, we used amplicon sequencing and potential function prediction on sediment and salt mat samples. We observed that the bacterial and fungal diversity in Lake Magic is strongly driven by carbon, temperature, pH and salt concentrations at the different stages of the lake. We also saw that the fungal diversity decreased as the environmental conditions became more extreme. However, prokaryotic diversity was very dynamic and bacteria dominated archaeal species, both in abundance and diversity, perhaps because bacteria better tolerate the extreme variation in conditions. Bacterial species diversity was the highest during early flooding stage and decreased during more stressful conditions. We observed an increase in acid tolerant and halotolerant species in the sediment, involved in functions such as sulfur and iron metabolism, i.e., species involved in buffering the external environment. Thus, due to activity within the microbial community, the environmental conditions in the sediment do not change to the same degree as conditions in the salt mat, resulting in the sediment becoming a safe haven for microbes, which are able to thrive during the extreme conditions of the evapo-concentration and desiccation stages.

## Introduction

Acid saline lakes represent one of the most extreme aquatic environments on Earth. They are poly-extreme ecosystems, exhibiting extremely acidic pH and salinities close to saturation. Such environments are of significant microbiological interest as they host organisms that are not only capable of withstanding pH and salinity stress, but also survive in the presence of additional stressors such as high metal concentrations and low nutrients (Mormile et al., 2007; Heidelberg et al., 2013; Johnson et al., 2015; Zaikova et al., 2018). Moreover, they serve as a reservoir of novel microbial functions such as acidophilic microorganisms that have been used for extracting metal ores from sulfide minerals (Dopson et al., 2017). Hence, such environments are of significant interest to scientists interested in understanding the microbial diversity dynamics of communities residing there, as well as applied areas developing microbial consortia for bioprocessing applications (Dopson et al., 2017).

Lake Magic is one of the most extreme acidic hypersaline lakes (ca. 1 km in diameter) present within the Yilgarn Craton in Western Australia. This unique lake exhibits extremely low pH (<1.6) coupled to very high salinity (32% TDS) with the highest concentration of aluminum (1,774 mg/L) and silica (510 mg/L) in the world (Bowen and Benison, 2009; Conner and Benison, 2013). Lake Magic, similar to other lakes in Western Australia, has dynamic and characteristic stages of lake transformation, including flooding, evapo-concentration and desiccation that are driven by the local seasons (Bowen and Benison, 2009; Conner and Benison, 2013). The lake is fed via both regional acidic groundwater and infrequent precipitation (Benison et al., 2007).

Recent studies of the microbial diversity of Lake Magic have revealed that the lake hosts acidophilic, acid-tolerant, halophilic, and halotolerant bacterial species (Zaikova et al., 2018). In addition, fluid inclusions of halite crystals from Lake Magic exhibited the presence of micro-algae and prokaryotes trapped within them (Conner and Benison, 2013) while metagenomic analyses of lake water, groundwater and within the sediment of Lake Magic revealed that the lake is dominated by only a few species, such as *Salinisphaera*, and has a low representation of other bacterial species (Zaikova et al., 2018). Interestingly, eukaryotes, including fungi and green algae, were abundant in the pelagic zone, giving rise to the bright yellow color of the lake (Conner and Benison, 2013; Zaikova et al., 2018). These studies provide indicators of the population residing within the lake and the functional niches they occupy. However, they do not examine how the microbial populations in the lake change during different transformational stages of the lake. Molecular approaches to understand the dynamics of Yilgarn Craton lakes in the past have focused primarily on spatial composition at a single timepoint (Mormile et al., 2009; Johnson et al., 2015; Zaikova et al., 2018; Aerts et al., 2019). In contrast, temporal approaches to study microbial community dynamics have revealed considerable variability within microbial community compositions over time in various studies (Ju and Zhang, 2014; Nagarkar et al., 2018; Chénard et al., 2019; Cruaud et al., 2019), showing that some taxa remain consistent in their abundance whilst others exhibit sudden blooms (Nagarkar et al., 2018).

We hypothesize that the large fluctuations in environmental parameters during lake transformations are key drivers which will lead to marked changes in microbial populations and contribute to the mechanisms driving the dynamics of these communities (Cruaud et al., 2019). Studying the temporal dynamics of microbial communities in poly-extreme ecosystems such as Lake Magic could reveal crucial information about the drivers of community diversity and stability (Nagarkar et al., 2018). This study attempts to investigate the diversity and potential functional dynamics of prokaryotic and fungal communities over a period of 1 year. Using 16S rRNA gene and ITS gene amplicon sequencing data generated for salt mat and sediment samples, we elucidate the diversity patterns generated during lake transformations and assess likely functional profile of the resident microorganisms.

## Materials and Methods

### Sample Collection

**FIGURE 1:**
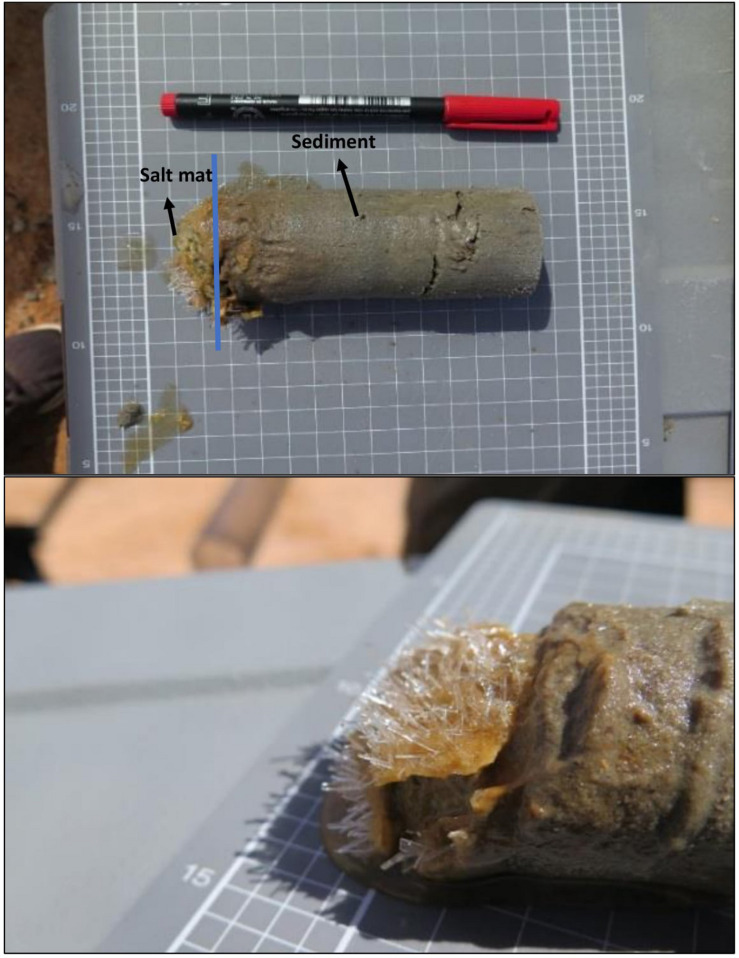
A single core of sample (∼6 cm deep) showing the salt mat and sediment sections. The bottom figure shows the salt mat (∼1 cm deep) with salt crystals on the top of sediment, sampled as a separate sample.

Core samples were collected from Lake Magic at timepoints across the flooding and evapo-concentration yearly cycle of the lake for 1 year (July 2017–July 2018). Multiple sampling sites were chosen around the lake for a single timepoint. Two types of samples were obtained from each core, namely a salt mat and a sediment sample (Figure 1). The salt mat layer changes physically during different stages of the lake, with the salt mat becoming more solid as the lake dries. With this in mind, the top 1 cm of the core was transferred to a clean falcon tube using a sterile spatula and regarded as the salt mat. The rest of the core was labeled as sediment (∼6 cm deep). All cores were taken with sterile core samplers and spatulas, and after every core the core samplers and spatulas were sterilized with 70% ethanol. A total of 5 sediment samples and 5 salt mat samples were collected. Temperature, pH, and salinity were measured for each sampling point using Orion 3 star Benchtop instrument. The pH and salinity were calibrated using standard solutions provided with the instrument kit. The pH and EC were measured in 1:5 sample-water extract. All samples were kept frozen at −20°C in 50 ml Falcon tubes in a portable freezer and moved to the laboratory freezer (−20°C) until further processing.

### DNA Extraction, PCR and Amplicon Sequencing

DNA from sediment and salt mat samples was extracted using a method developed for acid saline sediments. Briefly, 0.4 *g* sediment and salt mat samples were taken in 2 ml tubes containing glass beads. To this 900 μl of extraction buffer (consisting of 0.2 M sodium phosphate buffer, 0.2% CTAB, 0.1 M NaCl, and 50 mM EDTA), 100 μl of 10% SDS and 10 μl of Proteinase K (20 mg/ml) were added. The mixture was kept at -80°C for 5 min and then heated at 70°C for 20 min. The mixture was subjected to mechanical agitation at 20 Hz for 20 min after which the tubes were kept on ice for 5 min. After bead beating the mixture was centrifuged at 10,000 × *g* for 5 min. To the supernatant 750 μl of chilled phenol-chloroform-isoamyl (pH 8) solution was added and centrifuged for 20 min at 10,000 × *g*. Aqueous layer was transferred to sterile tube and 600 μl of chilled chloroform -isoamyl (pH 8) solution was added. The mixture was centrifuged at 10,000 × *g* for 5 min. Next, to the aqueous layer 650 μl of 20% PEG, 2.5 M NaCl was added and incubated at 4°C for overnight. The solution was centrifuged for 20 min at 10,000 × *g*. The obtained pellet was washed with 70% ice cold ethanol and 2 μl glycogen. The final pellet was dissolved in 50 μl of TE buffer. All extractions were carried out in triplicate. Tubes containing no sample were incorporated as extraction blanks (controls) and were treated identical to sample extractions. DNA concentration was measured through fluorometry using a Qubit dsDNA HS Assay Kit with a Qubit 2.0 fluorometer (Life Technologies).

Extracted DNA from all timepoints were diluted 10-fold prior to PCR amplification of the 16S rRNA and ITS genes. All PCR reactions were carried out in triplicate. PCR amplification of the 16S rRNA gene V4-V5 region was performed using the universal PCR primer set 515F and 806R, targeting members within both bacterial and archaeal domains (Whiteley et al., 2012). The forward primer included the addition of an Ion Torrent PGM sequencing adapter, a GT spacer and a unique Golay barcode to facilitate multiplexed sequencing. Barcoded PCR reaction mixtures (20 μl) consisted of DNA template (1 μl), universal primer mix (untagged 515F and 806R at a final concentration of 0.2 μM), tagged 515F primer (0.2 μM), 600 ng BSA (Life Technologies) and 2.5 × 5′Hot Master Mix (5Primer, Australia). The PCR cycle was set at 94°C for 2 min followed by 25 cycles of 94 min for 45 s, 50°C for 60 s, and 65°C for 90 s. This was followed by 2 cycles of 94°C for 45 s, 65°C for 90 s and final extension at 65°C for 10 min.

Amplification of the fungal ITS regions was carried out using the universal primer set ITS1 F and ITS2 R (White et al., 1990; Gardes and Bruns, 1993), with the addition of an Ion Torrent PGM sequencing adapter, a GT spacer and a unique Golay barcode to the forward primer. The barcoded PCR primer mixtures (20 μl) included DNA template (1 μl), universal primer mix (untagged ITS1 F and ITS2 R at a final concentration of 0.2 μM), 600 ng BSA (Life Technologies), tagged ITS1 F primer (0.2 μM) and 2.5 × 5′ Hot Master Mix (5Primer, Australia). The PCR conditions included initial denaturation at 94°C for 2 min followed by 25 cycles of 94°C for 45 s, 50°C for 60 s, and 65°C for 90 s. This was followed by 9 cycles of 94°C for 45 s, 65°C for 90 s, and a final extension stage at 65°C for 10 min.

Several positive, no template (as negative controls) controls and DNA extraction controls (extraction blanks) were amplified along with the samples for both bacterial and fungal marker genes. PCR reaction performance was checked by loading PCR amplicons along with positive and negative controls on a 2% (w/v) agarose gel. The amplicons were quantified using a Qubit dsDNA HS Assay Kit on the Qubit 2.0 fluorometer (Life Technologies). All amplicons were subsequently pooled in one composite mixture at a concentration of 20 ng/μl, including negative controls. The pool was purified using AMPure XP (Beckman Coulter, Australia) and the quality of the pool was checked by visualizing on a 2% (w/v) agarose gel. The composite pool was sequenced on an Ion Torrent PGM.

### Sequence Analysis and Statistical Analysis

Raw sequences were de-multiplexed and quality filtered through a custom QIIME version 1 pipeline (Quantitative Insights into Microbial Ecology; Caporaso et al., 2010) with a minimum average quality score of 20. The minimum sequence length was maintained at 130 b.p. and maximum sequence length of 350 b.p. Chimeric sequences were removed using USEARCH v6.1. No forward or reverse primer mismatches or barcode errors were allowed and maximum sequence homopolymers allowed were 15. The maximum number of ambiguous bases was set at six. *De novo* OTU picking was performed using ULCUST at 97% sequence identity cut off values and taxonomy was assigned through the Greengenes database (version 13.8). For fungal data, taxonomy was assigned using the SILVA v123 database (Quast et al., 2012).

The OTUs tables obtained for different levels of taxonomy were used as measures of taxa relative abundance in univariate statistical analysis. The OTUs detected in negative controls were manually removed from the data set. Alpha (α)- diversity at the phylum level for both 16S rRNA gene and ITS gene data was calculated using the richness, evenness and Shannon Weiner diversity index using the relative frequency table generated from a rarefied BIOM table. Data normality was checked with the Shapiro–Wilk test and log transformations of the data were performed where appropriate. Differences in the diversity for each stage and layer (sediment, salt mat), was calculated using a two-way analysis of variance (ANOVA). Tukey’s HSD *post hoc* comparisons of groups were used to identify which groups were significantly different from each other.

Beta diversity (β) of microbial communities was calculated with non-metric multidimensional scaling (NMDS) using Bray-Curtis dissimilarity (Bray and Curtis, 1957) of OTUs table. Statistical significances of dissimilarity, based on temporal data and sample layer, was assessed using main effect and pairwise ANOSIM in R using the vegan package.

Phylogeny was inferred using Phylosift (Darling et al., 2014) for sequences that could not be classified past the domain level. The OTUs abundance and diversity patterns were calculated using the vegan package (Dixon, 2003) in R software (R Core Team, 2014). Plots and heat maps were produced using “ggplot2” (Wickham, 2011), ggpubr packages in R and the online suite Calypso (Zakrzewski et al., 2017).

Predicted microbial functions of the bacteria residing within Lake Magic were generated using FAPROTAX, using default settings. FAPROTAX is a manually constructed database that maps the microbial taxa to metabolic functions (Louca et al., 2016). The output from FAPROTAX was visualized in R using the ggplot2 package.

### Chemical Analysis

Ten grams of sediment sample for each time point was oven dried at 60°C until completely desiccated. The sample was crushed, sieved to 2 mm fractions, packed in plastic bags and sent to the School of Agriculture and Environment, University of Western Australia chemical analysis laboratory. The analytical analysis included analysis of phosphorus, potassium, sulfur, organic carbon, nitrogen, iron, copper, sodium, boron, calcium, zinc, aluminum, magnesium, and manganese. These were extracted using Mehlich-3 extraction analyzed by inductively coupled plasma optical emission spectroscopy. Concentrations were expressed as percentage per weight and mg/kg.

## Results

### Lake Stages and Physico-Chemical Parameters

**FIGURE 2:**
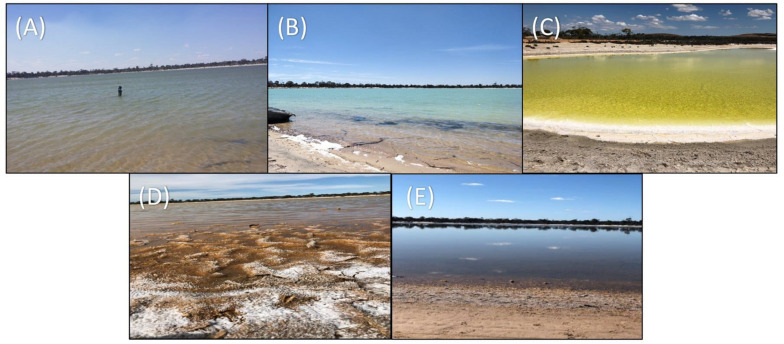
Five stages of Lake Magic in a span of 1-year **(A)** July 2017, flooded **(B)** October 2017, early evapo-concentration **(C)** January 2018, mid evapo-concentration **(D)** March 2018, and late evapo-concentration **(E)** July 2018, early flooding.

A total of 5 time points representing 5 different stages of Lake Magic were sampled for this study. Although the lake did not go through some of the more extreme physical changes during the year, the transformation of one stage to the next was evident. Namely, we observed flooding, early evapo-concentration, mid evapo-concentration, late evapo-concentration and early flooding in the lake (Figure 2) details of which are presented in Table 1 and Table 2. During the span of this study the lake did not reach complete desiccation due to heavy rainfall from 2017 to 2018 in Western Australia.

**TABLE 1:** Description of sampling site at different sampling time points for the water and sediment samples.

Lake stage	Sample code	Date collected	pH	EC(mS/cm)	Temperature (°C)	Lake description
Flooded	FL	July 2017	4.5 sediment 4.2 water	7 sediment 73.5 water	16	Clear blue lake water, very thin salt mat layer
Early evapo-concentration	EE	October 2017	4.5 sediment 4.1 water	11.3 sediment 78.3 water	25	Shallow blue water, salt mat becomes more evident
Mid evapo-concentration	ME	January 2018	4.5 sediment 3.4 water	15.95 sediment 146.9 water	28	Yellow slime water, salt foams forms on the lakeshore, thick salt mat layer develops, strong pungent smell in the air
Late evapo-concentration	LE	March 2018	4.2 sediment 2.7 water	41.8 sediment 223.8 water	33	A thick layer of salt mat with visible halite precipitation as crystals
Early flooding	EF	July 2018	4.8 sediment 3.41 water	55.9 sediment 226.7 water	24	Salt mat intact, water fills the dry lake bed, clear blue water

**TABLE 2:** Chemical data for samples at different time points.

Stage	Sampling time	% Carbon	% Nitrogen	Al (mg/kg)	B (mg/kg)	Ca (mg/kg)	Cu (mg/kg)	Fe (mg/kg)	K (mg/kg)	Mg (mg/kg)	Mn (mg/kg)	Na (mg/kg)	P (mg/kg)	S (mg/kg)	Zn (mg/kg)
Flooded	July 2017	1.01	0.062	1,007	3.7	350	0.8	64	323	587	0.6	7,056	6	488	0.1
Early evapo-concentration	October 2017	0.597	0.061	1,023	3.6	11,308	0.4	47	357	733	0.9	9,526	10	8,716	0.1
Mid evapo-concentration	January 2018	1.30	0.088	1,256	5.7	2,750	0.6	52	598	1,462	1.5	19,260	12	2,591	0.2
Late evapo-concentration	March 2018	0.739	0.085	1,099	7.5	20,565	0.6	77	1,065	2,750	2.5	37,861	8	16,261	0.2
Early flooding	July 2018	1.56	0.064	1,186	6.8	10,790	0.7	33	781	2,661	1.5	33,759	7.4	9,190	0.2

*All values shown are assessed using a single sample at each time point.*

The first sampling point was during late winter to early summer in 2017, where the lake was filled with several centimeters of clear blue water (July 2017; Figure 2A). The lake sediment was rich in clay and a small amount of wet salt mat sample was acquired. In October 2017, the lake became shallower (Figure 2B) during early evapo-concentration (Table 1), characterized by clear water but where halite precipitation was evident on the lake shore. The lake transformed into a shallow yellow lake during January 2018 sampling (Figure 2C) where the surroundings of the lake were rich in halite precipitation, the lake itself exhibited a pungent acidic odor and the salt mat became desiccated and substantial. During mid-summer (March 2018) the lakebed became dry and a thick salt crust was observed on the surface of the salt mat (Figure 2D) with visible salt crystals (Figure 1), constituting the late evapo-concentration stage. At this stage the lake was also rich in iron oxide precipitation, which was evident due to its distinct red color of the sediment. The final sampling timepoint was the beginning of the flooding stage in July 2018 (Figure 2E) where the lake started to fill with water, with a concomitant dissolving of the halite and iron precipitation observed during the evapo-concentration stages. The chemical data has been summarized in Table 2.

### Temporal Dynamics of the Lake Magic Microbiome

In order to assess the microbial community dynamics in the lake at different stages, we used 16S rRNA gene and ITS gene Ion Torrent sequencing (15 Salt mats; 15 Sediments) in triplicate. After DNA extraction, all samples had detectable amounts of DNA, however, the DNA concentration was consistently higher for salt mat samples when compared to the sediment samples. Statistical analysis, however, showed that there was no significant difference (ANOVA *p* > 0.05) between diversity and richness indices of salt mat and sediment layers for both bacteria and fungi analyses (Supplementary Figure 1). Generally, we found DNA extraction easier from salt mat samples, relative to sediment samples, due to the lower amount of clay present in the salt mat samples.

A total of 330,913 reads were obtained for 16S rRNA gene microbiome analyses, representing 15,947 OTUs. The OTUs were assigned to 37 phyla, 135 classes, 260 orders, 426 families, and 739 genera of archaea and bacteria. Only OTUs which appeared in sequenced negative controls were discounted from the analyses and no other OTUs were filtered. This is because low abundance OTUs can have a significant effect on the diversity metrics for the microbial communities in low biomass environments, hence, filtering of OTUs with low representation in the microbiome can result in loss of crucial information. Analysis of the ITS gene sequences revealed a total of 724,490 reads, representing 4,005 OTUs and were assigned to 15 phyla, 44 classes, 86 order, 177 family and 259 genera.

### Bacterial Communities Are Dynamic in Lake Magic

**FIGURE 3:**
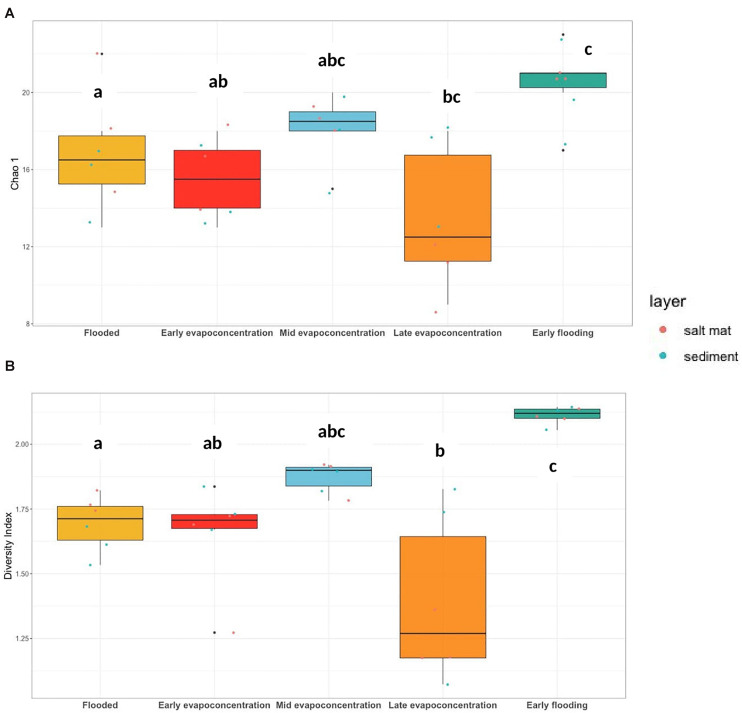
**(A)** 16S rRNA gene OTUs richness, Chao 1 (ANOVA, *p* = 0.001) and **(B)** Shannon-Weiner diversity index (ANOVA, *p* < 0.001) at phylum level. The letters show significant differences as obtained with *Tukey’s post hoc* test.

The Lake Magic microbiome OTUs richness was analyzed for salt mat and sediment samples on a relative frequency table constructed from a rarefied BIOM table (Figure 3A) at the phylum level which varied significantly between the five stages of the lake (ANOVA *p* = 0.001). The Chao 1 estimate varied from 9 to 23 for the 16S rRNA gene and the OTUs richness was significantly higher during the early flooding stage (mean richness = 21). The second highest OTUs richness was observed during the mid evapo-concentration stage (mean richness = 18). The late evapo-concentration stage showed the lowest mean richness of 13.6, whereas OTUs richness at the flooding stage was significantly different and showed a mean richness of 16. A mean richness of 15.5 was seen during the early evapo-concentration stage.

The Lake Magic microbiome diversity was also analyzed using a rarefied BIOM table at the phylum level. The diversity varied during the different lake stages (Figure 3B) and followed a similar trend to that of OTUs richness. The Shannon-Weiner diversity index ranged from 1.61 to 2.13 for bacterial communities, where the diversity was seen to increase during early evapo-concentration (mean diversity = 1.65) and the mid evapo-concentration stage (mean diversity = 1.87). However, the diversity decreased during the late evapo-concentration stage and was recorded as the lowest diversity index (mean diversity = 1.39) of all stages. The highest diversity was seen during the early flooding stage (mean diversity = 2.11). The ANOVA test was further analyzed with a *Tukey’s post hoc test* for diversity and richness indices, which revealed that the diversity during flooding, late evapo-concentration and early flooding stages were significantly different from each other, whereas the richness was significantly different only during flooding and early flooding stages.

The Lake Magic bacterial composition under different lake stages was analyzed and is shown in Supplementary Figure 2. Archaeal community diversity in the microbiome was represented by only two phyla, the *Crenarchaeota* and the *Euryarchaeota*, whilst the bacterial domain contributed the dominant OTUs observed within the 16S rRNA gene analyses. The majority of the sequences for bacteria originated from two phyla: *Bacteriodetes* (20%) and *Proteobacteria* (39%), most of which could not be classified below family level, indicating that a large proportion of the bacterial taxa in Lake Magic appear to be relatively poorly characterized.

### The Bacterial Community Becomes More Specialized as Stress Increases

For taxa which could be classified to genera, varying trends were observed during different lake stages and within the sample layers (Supplementary Figure 3). Specifically, key bacterial genera fluctuated within the salt mat and sediment during the various lake stages. The correlation analysis of chemical data with the bacterial diversity revealed that microbial dynamics is strongly driven by salinity, temperature, pH and carbon content in the lake (Supplementary Figure 4). For instance, members of the *Acidiphilium* genus were low in abundance during the flooding stage in both the salt mat and sediment samples, whilst their relative abundance was seen to increase within the salt mat during evapo-concentration stage as the lake conditions became more stressful. A significant increase (ANOVA, *p* < 0.05) in *Acidiphilium* relative abundance was observed within the salt mat during the late evapo-concentration stage and significantly decreased during the early flooding stage of the lake (Supplementary Figure 3, *Acidiphilium*). Similarly, the relative abundance of *Acidobacterium* was high within sediments during the flooding stage and a significant increase in abundance within the salt mat was seen during all evapo-concentration stages (Supplementary Figure 3, *Acidobacterium*). Similar to *Acidiphilium*, the relative abundance of *Acidobacterium* decreased when the lake was in the early flooding stage. In contrast, sequences belonging to *Arthrobacter, Bacillus, Flavbacterium, and Sporosarcina* increased significantly in relative abundance during the early flooding stage in both the sediment and salt mat, whilst genera such as *Nitrososphaera* were only present during the early flooding stage in high relative abundance.

Interestingly, it was also observed (Supplementary Figure 3) that the relative abundance of *Sulfurimonas, Syntrophobacter, Halothiobacillus, Acidobacterium, Acidiphilium*, and *Alicyclobacillus* decreased during the early flooding stage in both the salt mat and the sediment, whilst, the *Syntrophobacter* population increased in relative abundance within the sediment during late evapo-concentration. During the flooding stage *Salinisphaera* was found to be more abundant in the sediment when compared to the salt mat. However, in the late evapo-concentration stage, its abundance increased in the salt mat (Supplementary Figure 3).

### The Fungal Community Becomes Less Diverse Under Extreme Conditions Within Lake Magic

**FIGURE 4:**
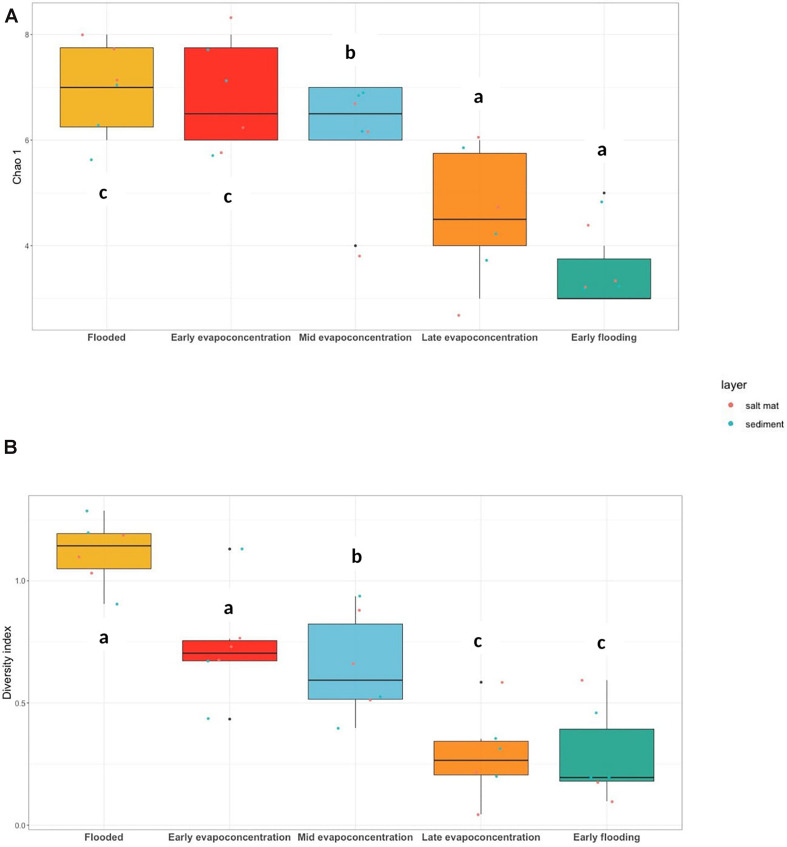
**(A)** ITS gene OTUs richness, Chao 1 (ANOVA, *p* = 0.001) and **(B)** Shannon-Weiner diversity index (ANOVA, *p* < 0.001) at phylum level. The letters show significant differences as obtained with *Tukey’s post hoc* test.

The diversity and richness of the fungal community within Lake Magic was analyzed similar to 16S rRNA gene analysis using the rarefied BIOM table at the phylum level. The diversity of fungi significantly decreased (ANOVA, *p* < 0.001) as the lake conditions became more extreme. The richness index ranged from 2 to 7 whereas the Shannon diversity index ranged from 0.004 to 1.28 (Figure 4A,B). The highest mean richness was observed for the flooding and early evapo-concentration stage. The OTUs richness consistently decreased after the early evapo-concentration stage, with lowest OTUs richness observed for the early flooding stage (mean richness = 2). The fungal diversity showed a varying trend when compared to OTUs richness, where the highest diversity index was observed for the flooding stage (mean diversity index = 1.11) whereas the lowest was observed for the extreme early flooding stage (mean diversity index = 0.28). A Tukey’s *post hoc* test revealed that the flooding and early evapo-concentration stage were statistically similar to each other, whilst, the middle evapo-concentration and late evapo-concentration stages were statistically similar, and the early flooding stage was significantly different from all other stages. Additionally, a Tukey’s *post hoc* test for OTUs richness showed that the flooding, early evapo-concentration and early flooding stages were significantly different from all other stages.

Interestingly, when the fungal community composition was visualized (Supplementary Figure 5), an increase in unidentified fungi belonging to the *Ascomycota* phylum was observed. This indicated that a large portion of the fungi living in Lake Magic are likely unidentified. The salt mat during the early flooding stage was, however, the most diverse when compared to other time points and was abundant with the genus *Cladosporium*. Variation in the composition of other genera including *Fusarium, Ulocladium*, and *Hostaea* was also seen.

### Comparing the Bacterial and Fungal Communities

**FIGURE 5:**
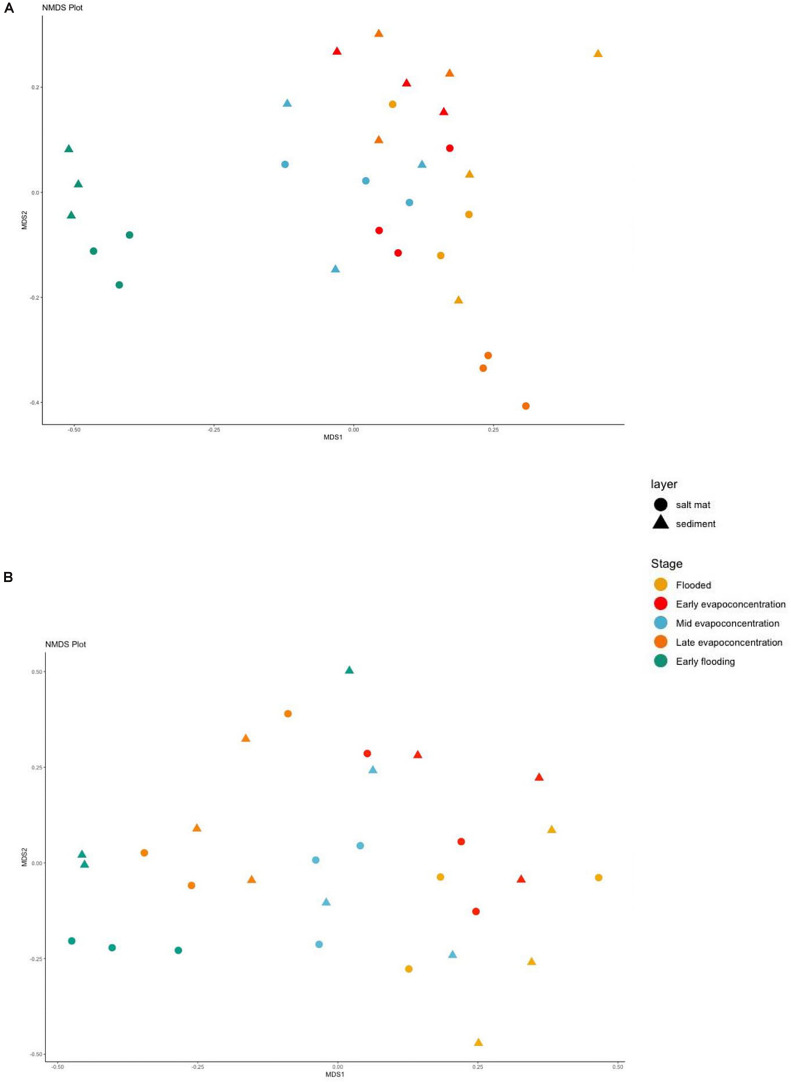
Beta diversity shown in a Non-metric multidimensional scaling (NMDS) plot using Bray-Curtis dissimilarity indices for **(A)** 16S rRNA **(B)** ITS sequences for different time points and layers at phylum level. Samples are colored according to the time points and shaped according to the layer.

The dissimilarity between microbial communities at different lake stages was assessed using NMDS using Bray–Curtis dissimilarity indices made with the OTUs table at phylum level. The sediment and salt mat bacterial communities from the flooding, mid evapo-concentration and late evapo-concentration stages tightly clustered together (Figure 5A). In contrast, the salt mat and sediment communities under the early flooding stage clustered separately (ANOSIM, *R*^2^ = 0.49, and *p* = 0.001). However, similar to alpha diversity, when an ANOSIM test was applied to determine the variability in the salt mat and sediment layers, no significant difference was observed (ANOSIM, *R*^2^ = 0.05, and *p* = 0.103).

The NMDS analysis of fungal communities showed less clustering when compared to the bacterial community analysis (Figure 5B), apart from the early flooding stage, where beta diversity of the fungal community was statistically different (ANOSIM, *R*^2^ = 0.462, and *p* = 0.001). However, no significant difference was found in the beta diversity of fungi in the sediment versus the salt mat layers.

Finally, it is interesting to directly compare the bacterial and archaeal communities’ abundances and diversities at the different time points (Supplementary Figures 2, 5, respectively). We see that, in general, the bacterial community was both more abundant and more diverse, with the exception of the early-flooding time point in the salt mat sample, where the diversities were similar, but the archaeal community was more abundant.

### Predicted Ecological Functions of the Bacterial Communities

**FIGURE 6:**
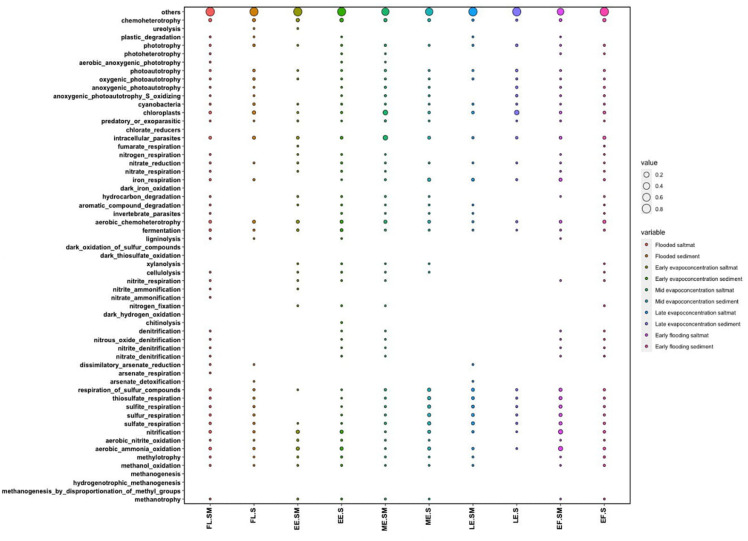
Dot plot showing the FAPROTAX predicted functions of the bacterial community within the salt mat and sediment samples at different lake stages.

The ecological functions of the bacterial communities were predicted using the FAPROTAX pipeline for the different stages of the lake. These analyses indicated aerobic chemoheterotrophy, iron oxidation and sulfur-related pathways as likely dominant functions within the lake’s bacterial community (Figure 6). Functions related to carbon metabolism, such as methylotrophy and methanotrophy were predicted but were not abundant. Nitrogen-related functions included nitrification and ammonia oxidation and were observed to fluctuate between different lake stages. For instance, these data indicated that nitrification and ammonia oxidation-predicted functions in the lake were significantly higher within the salt mat during the early flooding stage (Supplementary Figure 6). Interestingly, the nitrogen-related predicted functions were also high in the sediment samples during the early evapo-concentration and mid evapo-concentration lake stage. Sulfur-related predicted functions were lower during the early flooding stage, but significantly increased in the sediment layer during all evapo-concentration stages. Sulfur-related predicted function was found to be highest in the sediment during the early evapo-concentration stage. Predicted functions related to iron oxidation and reduction were relatively low at all timepoints, but significantly increased during the early evapo-concentration and late evapo-concentration stage within the sediment layer. Finally, iron-based respiration predicted pathways fluctuated more frequently when compared to predicted sulfur and nitrogen-related functions during all lake stages (Supplementary Figure 6).

## Discussion

Acid saline lakes in Western Australia host unique microorganisms that can be studied to understand life under extreme conditions, novel biogeochemical processes and new biotechnological avenues (Benison et al., 2007). In this study, the bacterial and fungal microbial community dynamics were studied using a temporal approach to resolve how an extreme lake microbiome changes during different stress phases due to changes in the physico-chemical properties of the habitat.

Previous studies on acidic hypersaline lakes in Western Australia revealed a high level of bacterial diversity within Lake Magic and other Western Australia lakes water (Mormile et al., 2009; Zaikova et al., 2018), but have relied upon single time (lake stage) sampling points. Our results indicate that the alpha (α)-diversity of the bacterial and fungal microbial communities differed significantly between the less extreme (flooding) stage and the more extreme (late evapo-concentration) stage. These differences indicated that the lake microbiome diversity is driven to a large degree by the high salt and low pH conditions within the lake (Podell et al., 2014). It has previously been reported for hypersaline environments that the ionic concentration in these environments hinders the solubility of oxygen, and hence, the oxygen concentration is very low in acid saline lakes (Sherwood et al., 1991). However, the majority of microorganisms isolated from hypersaline environments are aerobic heterotrophs that are also capable of fermentation (Dyall-Smith, 2009). Our results are also in line with these previous findings. Moreover, our results show that the number of OTUs in the salt mat layer were consistently higher in all samples. These results are also similar to the results obtained by Aerts et al. (2019) for four acid saline lakes in Western Australia, and suggest that the microbial community residing in the salt mat likely has increased access to light, water and oxygen. This is also explained by the availability of oxygen at the air/water interface, which exhibits an unequal distribution of oxygen between the salt mat and the sediment layer. Since the salt mat is directly in contact with the water column in the lake there is higher availability of oxygen locally (Podell et al., 2014). Comparing the microbial composition of the salt mat and sediment in Lake Magic also indicates that microorganisms more tolerant to high salt and pH conditions are selected within the salt mat, where the environmental conditions such as salinity and pH are harsher and more dynamic compared to those in the sediment layer.

The prokaryotic community at all stages of the lake cycle was dominated by halotolerant and acidophilic bacteria, whilst archaeal taxa were found in much lower abundances. Archaeal sequences were derived from only two phyla and these observations are in good agreement with Johnson et al. (2015) who observed low archaeal diversity within four acidic hypersaline lakes in Yilgarn Craton. It has been reported for Lake Tyrell in Victoria, Australia, that succession of different microorganisms is dependent upon the solutes present in the lakes (Podell et al., 2014). Hence, the variation in solute concentration in Lake Magic at different stages is likely driving the succession of archaea and bacteria. It could also be hypothesized that bacterial taxa outcompete archaeal taxa in these unique environments because of the dynamic nature of the Western Australian lakes, where environmental changes are frequent and, therefore, stress tolerant species are selected, as opposed to obligate archaeal extremophiles (Benison and Bowen, 2006). However, it should also be considered that low representation of archaeal sequences might be because of the primer sequences used in this study.

Organic carbon and nitrogen concentrations were found to be low at all lake stages and increased slightly during the flooded (FL) stages, in line with previous studies in similar lakes in Western Australia (Ruecker et al., 2016; Aerts et al., 2019). Similarly, phosphorus levels were also low in Lake Magic, as in other Western Australian lakes (Aerts et al., 2019), with phosphorus often being considered a limiting factor along with carbon and nitrogen for the growth of microorganisms (Elser et al., 2007). Microbiome analyses indicated an abundant community of heterotrophs within the lake and hence, low carbon, nitrogen and phosphorus are likely to be responsible for the low the biomass in these samples (Aerts et al., 2019). However, carbon and nitrogen levels were highest during the mid evapo-concentration and these increases are likely due to photosynthetic inputs via blooming of the halotolerant alga *Dunaliella.* This is also reflected in the increased microbial diversity during the mid evapo-concentration stage. Blooming of algae commonly occurs during this lake stage and provides a source of photosynthetically derived nutrient input which causes increased diversity in the lake (Zaikova et al., 2018).

During the evapo-concentration stages iron concentrations increased which was also mirrored in the predicted microbiome functions of increased iron respiration activity within the bacterial community. Iron metabolism is considered an important function of the sediment inhabitants and is thought to be responsible for the increasing of pH in the sediment that is, becoming less acidic (Lu et al., 2016; Zaikova et al., 2018). The members of *Alicyclobacillus* genus are reported to be involved in iron oxidation along with archaea in other acid saline lakes in Western Australia (Johnson et al., 2015; Lu et al., 2016). The members of the genus are also capable of reducing iron (Yahya et al., 2008; Lu et al., 2010). Previous metagenomic studies of lake magic indicated that *Alicyclobacillus* was the most abundant genus within the sediment. Moreover, the genus *Acidiphilium* was also found to be abundant in the sediment samples (Zaikova et al., 2018) with members of this genus are involved in iron reduction (Weber et al., 2006; Sánchez-Andrea et al., 2011; Zaikova et al., 2018). These data indicated that the relative abundance of the members of *Alicyclobacillus* and *Acidiphilium* varied across different lake stages and is likely more complicated than first thought, in terms of lake biogeochemistry (Zaikova et al., 2018). *Alicyclobacillus* abundance was significantly higher in sediment samples during the flooding stage and was significantly increased during the early evapo-concentration stage within the salt mat samples. In contrast the genus *Acidiphilium* was more abundant within the salt mat samples during the later, late evapo-concentration stage. Interestingly the overall abundance of these genera decreased during the higher stress stages of the lake.

Previously it has been suggested that archaea dominate iron oxidation in acid saline extreme environment and that the role of bacteria is limited. Moreover, the iron activity is known to decrease by the presence of high salt in the environment (Lu et al., 2016). In the study, *Acidiphilium* spp. were isolated from an acid saline lake in Western Australia which formed long filamentous structures during low pH and high salinity conditions indicating that these species have developed a coping mechanism for extreme stress (Lu et al., 2016). Examining the functional profile of our samples it can be seen that predicted iron respiration increases significantly in the sediment during the early evapo-concentration and late evapo-concentration stage and is the lowest in the sediment during the flooding stage potentially because of the coping mechanism adapted by these species.

The activity of acidophiles is known to decrease significantly in presence of high concentration of chloride ions (Suzuki et al., 1999; Shiers et al., 2005). However, Lu et al. (2016) found that *Acidiphilium* spp. formed long filaments as a coping mechanism to high chloride ions within the Dalyup River in Western Australia. As iron metabolism is affected by the presence of chloride ions we hypothesize that this may explain the bacterial spp. of *Alicyclobacillus* and *Acidiphilium* being dominant during early evapo-concentration and flooding stages. It is also suggested that under the high chloride conditions, based upon community data, these species are responsible of increasing the pH in the sediment. Hence, we postulate from these results that microorganisms in Lake Magic adapt to stressful conditions of pH and salinity not only physiologically, but also play crucial roles in changing their external environment making it more habitable for themselves and other members of the lake.

One of the most abundant genera detected in these data was *Salinisphaera* (7%), in both the sediment and salt mat. *Salinisphaera* species are mesophilic, halotolerant and slightly acidophilic (pH range 5.0–7.5), surviving in a range of moderately acidic and saline conditions as well as high concentrations of metal ions. Species belonging to genus *Salinisphaera* have been isolated from a range of environments, including hydrothermal vents, solar salterns, brine from salt wells, seawater and marine fish surfaces (Antunes et al., 2003; Mormile et al., 2007; Crespo-Medina et al., 2009; Bae et al., 2010; Gi et al., 2010; Park et al., 2012; Zhang et al., 2012; Shimane et al., 2013). Critically, these species are able to metabolize both autotrophically and heterotrophically (Antunes et al., 2003, 2011; Crespo-Medina et al., 2009; Bae et al., 2010; Park et al., 2012; Zhang et al., 2012; Shimane et al., 2013). In addition, *Salinisphaera* species are also involved in the uptake of iron and siderophore production (Antunes et al., 2003). Molecular studies here indicated that the abundance of *Salinisphaera* was consistently high in salt mats during all lake stages, except during the FL stage, where it was more abundant within the sediment. *Salinisphaera* was previously reported to be the single dominant OTUs in the lake water (Zaikova et al., 2018) during evapo-concentration in Lake Magic. These findings together indicate that most of the *Salinisphaera* spp. reside in the water column of Lake Magic. The dramatic increase in its representation during the late evapo-concentration stage in the salt mat and sediment suggests that it is highly tolerant of extreme pH and acidic environmental conditions, including tolerance to heavy metal ions, allowing it to survive through the evapo-concentration stages.

Members of genus *Sulfurimonas* have been isolated from diverse environments such as hydrothermal vents, marine sediments and terrestrial habitats and are known to play an important role in chemoautotrophic processes (Han and Perner, 2015). The members of this genus can grow on a variety of electron donors and acceptors and, thus, are able to colonize disparate environments. These include different reduced sulfur compounds such as sulfide, elemental sulfur, sulfite, and thiosulfate (Han and Perner, 2015). Many members of the genus are also involved in nitrogen and hydrogen metabolism. In our samples, *Sulfurimonas* (1.2%) relative abundance continuously decreased in salt mats but increased in relative abundance within the sediments as the environmental conditions became more stressful. Ultimately, it decreased significantly during the most extreme (late evapo-concentration) lake stages but still maintained a low representation in the sediment. When examining the functional profile data, predicted sulfur functions significantly increased in the sediment samples during the evapo-concentration stages, suggesting that *Sulfurimonas* spp. play a crucial role in cycling key nutrients in the sediment. Since the members of the genus are able to survive chemolithoautotrophically using various electron acceptors and donors (Campbell et al., 2006; Grote et al., 2008), it suggests that these species are capable of adapting to the changing environmental conditions of Lake Magic. A similar trend of abundance was seen for *Flavobacterium, Bacillus, and Syntrophobacter*, where their abundances increased in the sediment during the evapo-concentration stages and we postulate that they play a crucial role in niche construction by interacting with other dominant taxa as the stress increases.

The fungal diversity was seen to decrease as the environmental conditions became more acidic and saline. A single phylum, *Ascomycota* (76.8%), became dominant as the lake became dry and hypersaline. Most of the members of this phylum are unidentified, and hence, most of the fungi residing in Lake Magic are either novel, or the sequence length of the marker gene used in this study is not sufficient to be able to characterize these members. It can be deduced that these fungi are tolerant of the acidic hypersaline conditions of the lake. In a previous study on Lake Magic microbiology, the water samples were found to be highly diverse in terms of eukaryotic community composition, being abundant (∼98.5%) in Ascomycota. *Aspergillus* and *Penicillium* were the most abundant genera in Lake Magic water. However, the results for sediment samples were quite similar to our results (Zaikova et al., 2018). The presence of the halotolerant algae *Dunaliella* during evapo-concentration stages is a major contributor to carbon content in the lake. It can be deduced from the data that as *Dunaliella* increase in the lake (during middle evapo-concentration) the diversity in the salt mat increases rapidly. Interestingly, this difference in the diversity of lake water and sediment column indicates the exchange between the two compartments in terms of nutrition. The water column is more abundant in oxygen and has access to sunlight whilst, the higher composition of eukaryotes in the water column which contribute to the carbon and nitrogen content in the salt mat and the sediment.

In conclusion, the paper describes the temporal diversity of life living in a unique poly-extreme environment. Specifically, we studied the microbiome of one of the most extreme environments on earth, having a pH of about 1.5 and salinity seven times that of sea water. These environments are highly under-studied. Moreover, the temporal dynamics of poly-extreme environments have not previously been investigated. The findings in this paper highlight many potential survival strategies of microbes living in a poly-extremophile environment. The results of the study also point out the role of microbial interactions as a survival strategy of microbes in such an environment (Zengler and Zaramela, 2018). Finally, we found previously unknown bacterial species that are found in these environments and need to be investigated further.

## Data Availability Statement

The datasets presented in this study can be found in online repositories. The names of the repository/repositories and accession number(s) can be found below: NCBI under the project ID PRJNA721776.

## Author Contributions

N-U-HG conducted field sampling, carried out the research, analyzed, and wrote the manuscript. MW and AW designed the study and wrote the manuscript. All authors reviewed and edited the manuscript.

## Conflict of Interest

The authors declare that the research was conducted in the absence of any commercial or financial relationships that could be construed as a potential conflict of interest.
