# Supplementary material for: Temporal Microbial Community Dynamics Within a Unique Acid Saline Lake
Source: Front Microbiol. 2021 Jun 24;12:649594. doi: 10.3389/fmicb.2021.649594 (PMC8264302; doi:10.3389/fmicb.2021.649594)
Supplement: Supplementary file 1 [file Data_Sheet_1.PDF]

Supplementary File

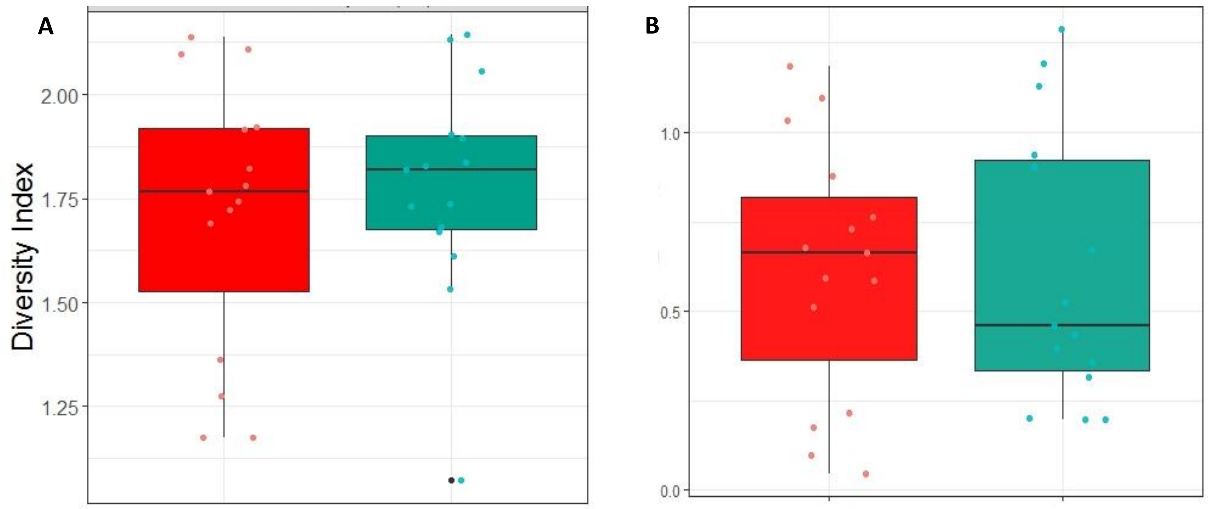

Supplementary Figure 1: (A) Shannon Weiner diversity index of 16S rRNA gene and **(B)** ITS gene between salt mat and sediment layers at phylum level. No significant difference was seen for diversity between the layers (ANOVA, p>0.05). Colours represents: Salt mat Sediment

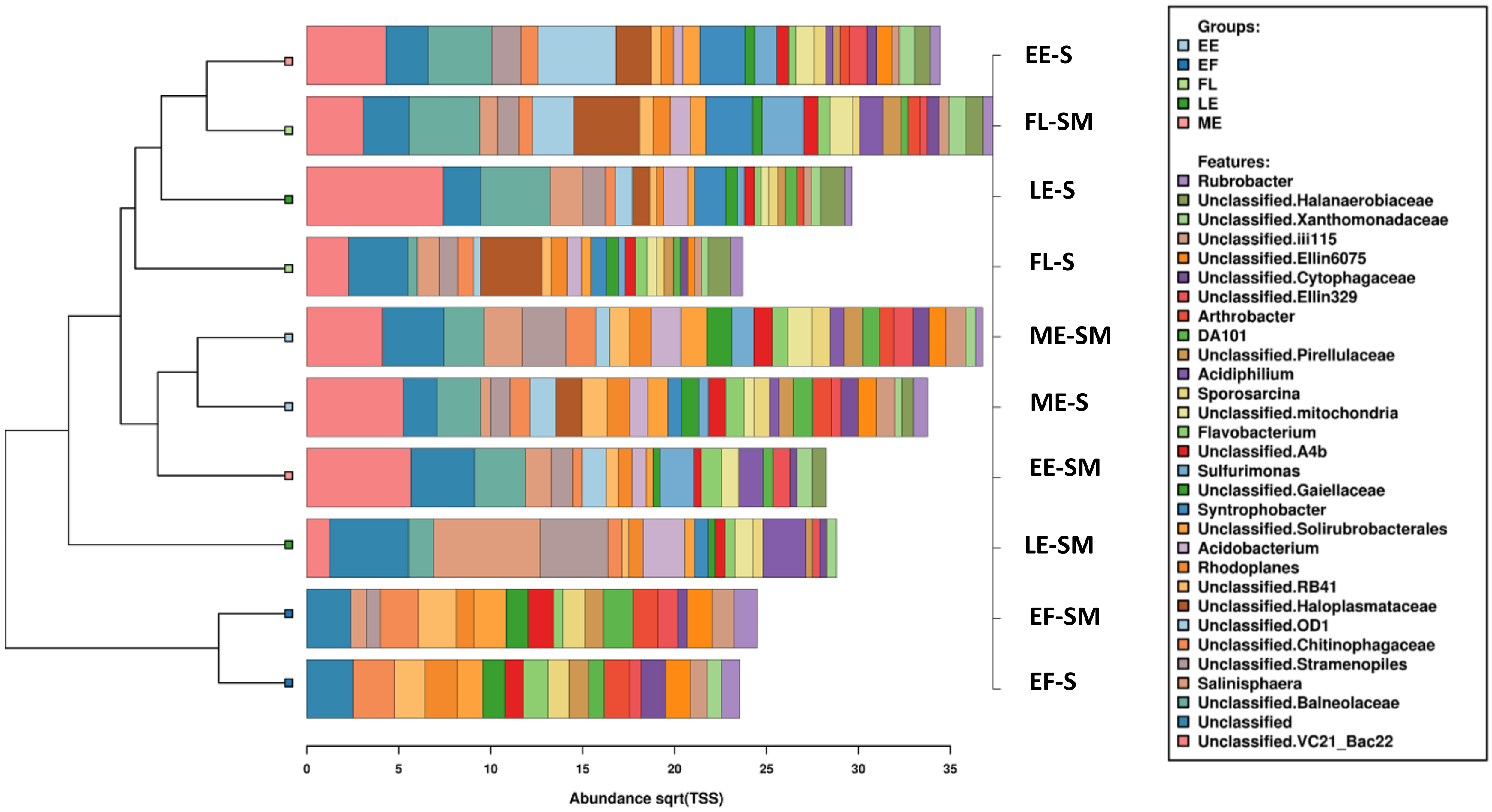

Supplementary Figure 2: Bar chart showing bacterial composition at genera level for each sample layer at different time points. The samples have been arranged so more closely related are clustered together.

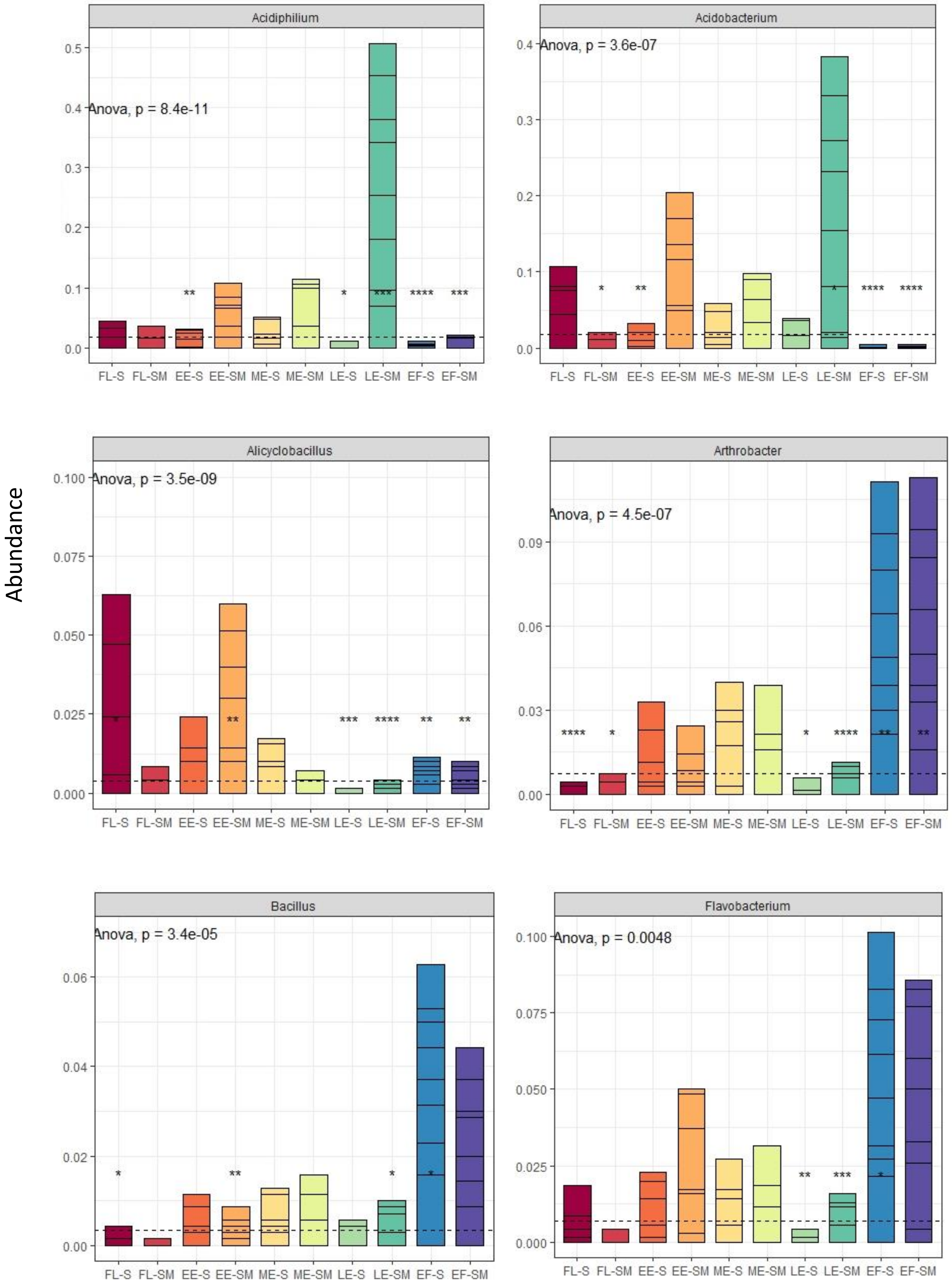
Abundance
Acidiphilium
Anova, p = 8.4e-11
Acidobacterium
Anova, p = 3.6e-07
Alicyclobacillus
Anova, p = 3.5e-09
Arthrobacter
Anova, p = 4.5e-07
Bacillus
Anova, p = 3.4e-05
Flavobacterium
Anova, p = 0.0048
FL-S FL-SM EE-S EE-SM ME-S ME-SM LE-S LE-SM EF-S EF-SM

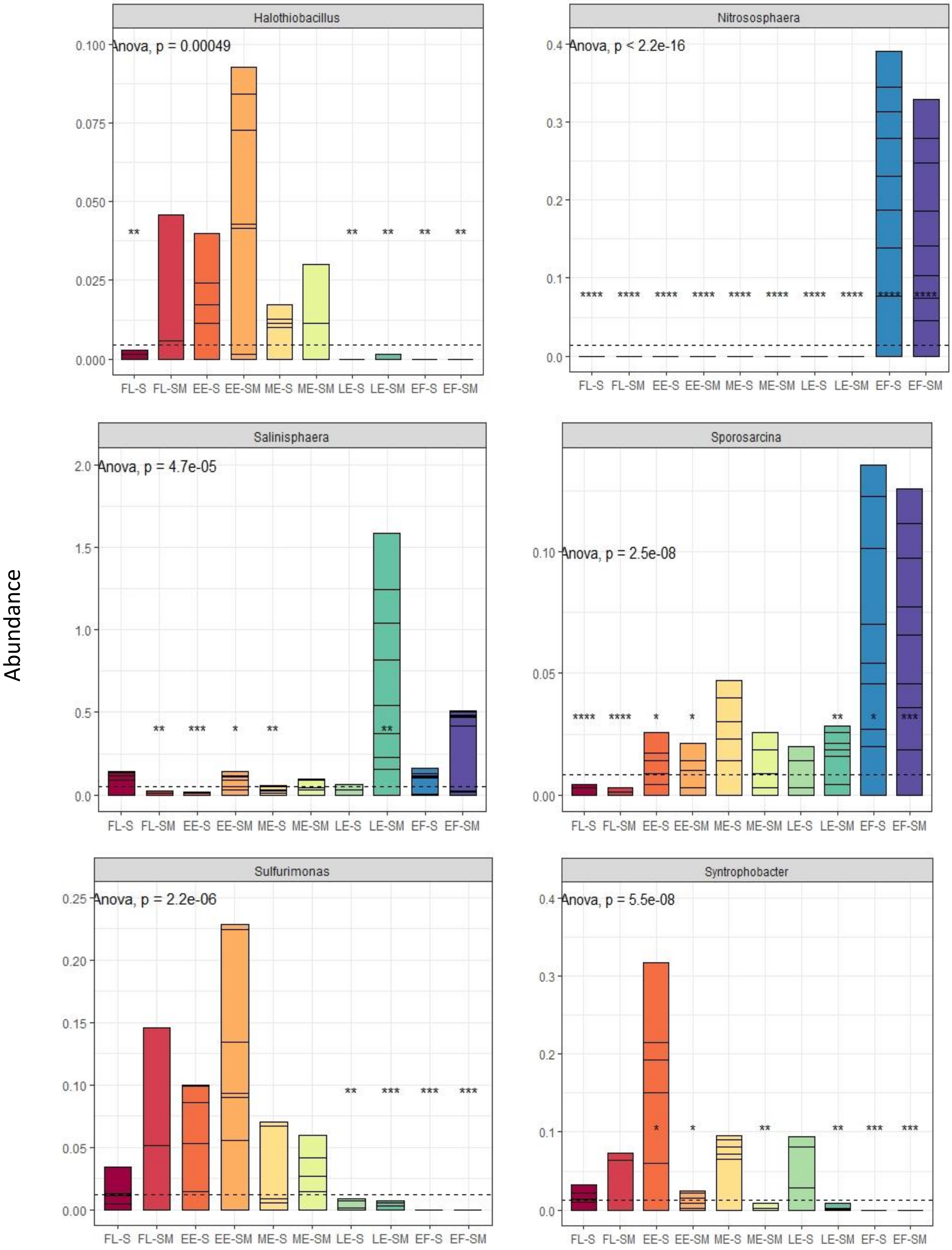

Supplementary Figure 3: Abundance of key genera during different stages and sample layers. Statistical significance is shown as asterisks (ANOVA).

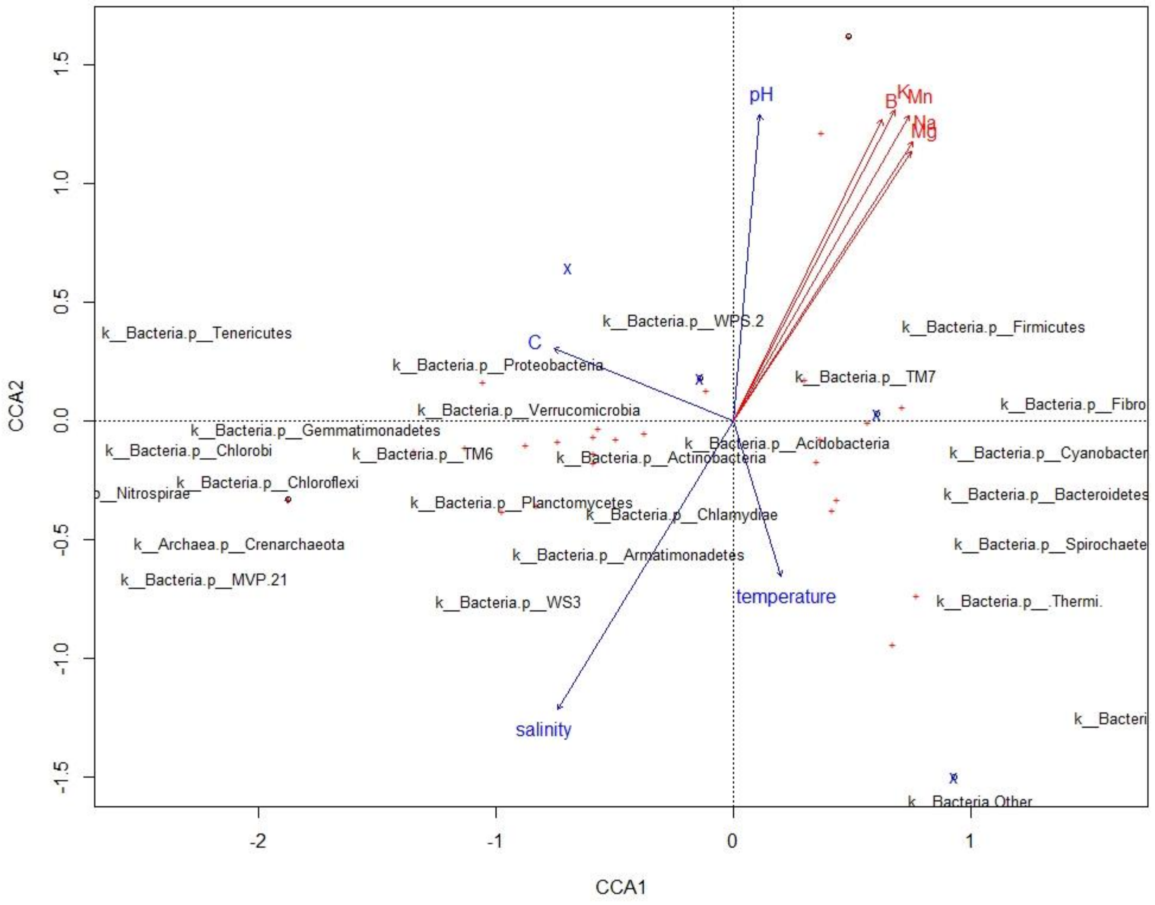

Supplementary Figure 4: CCA plot showing the relation of significant environmental factors with bacterial phylum. The blue arrows show statistically significant environmental factors. Red arrows show the remaining statistically insignificant environmental factors.

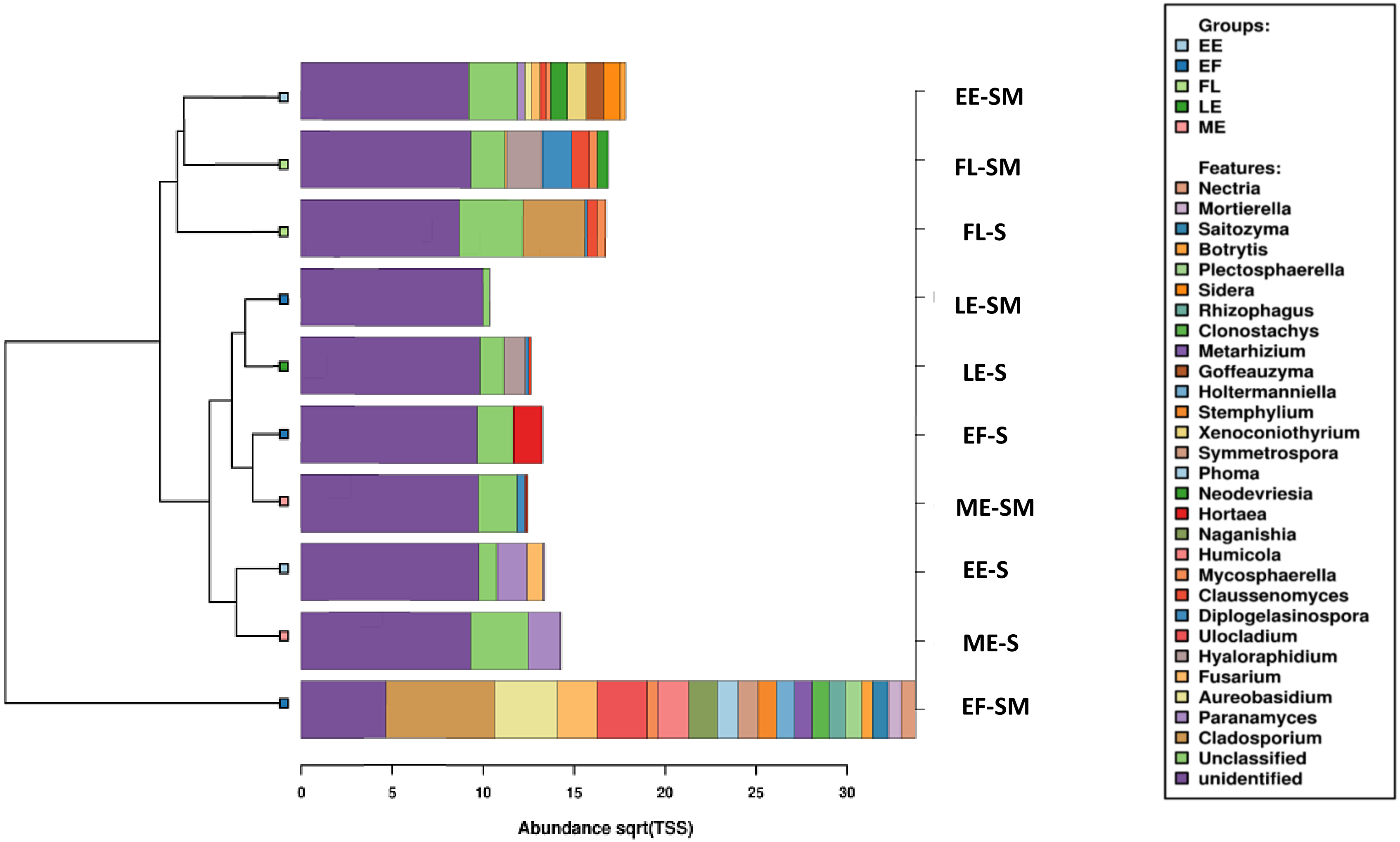

Supplementary Figure 5: Bar chart showing fungal composition at genera level for each layer at different time points. The samples have been arranged so more closely related are placed together.

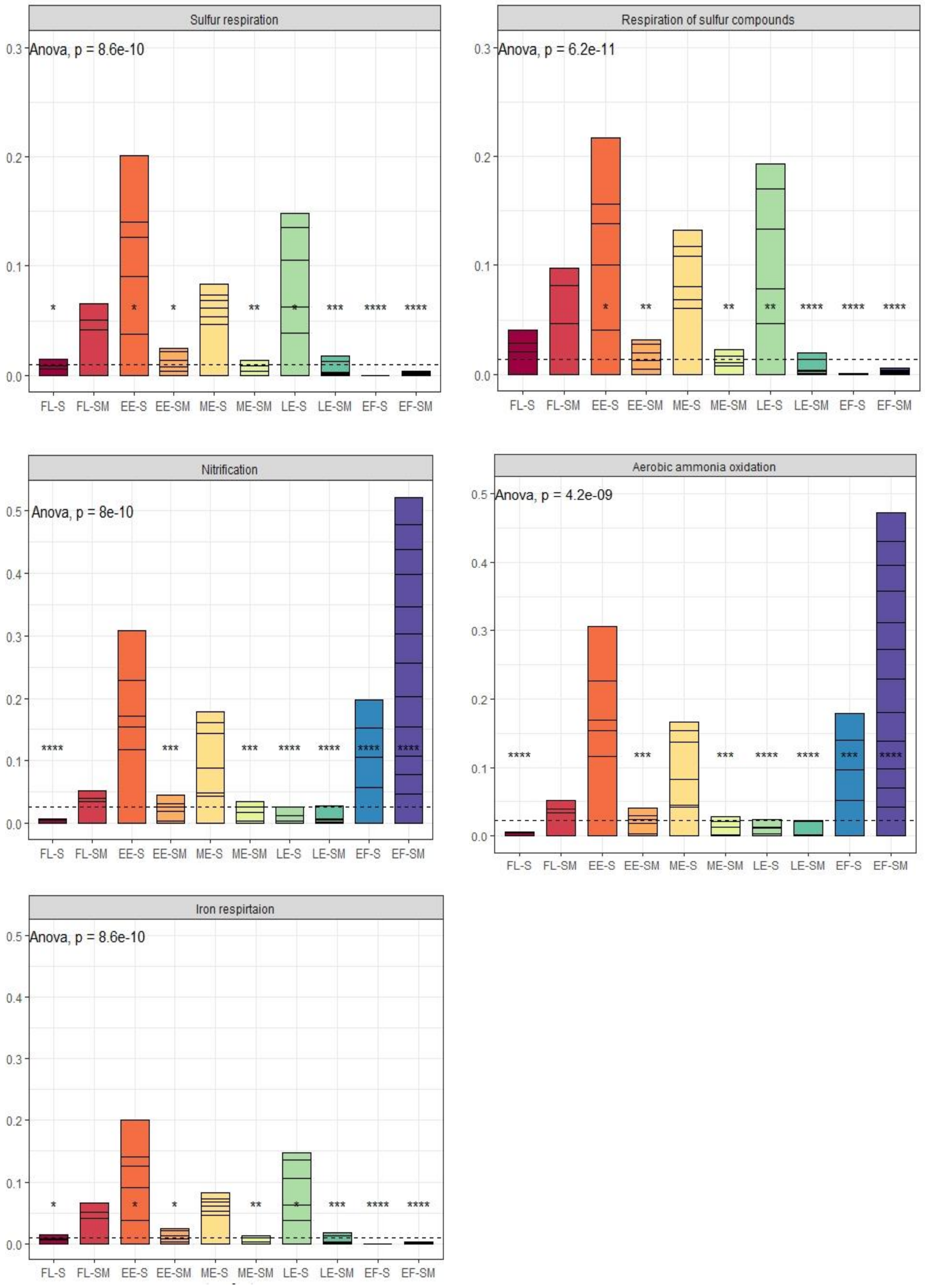

Supplementary Figure 6: Bar plots of individual functions of bacteria in the sediment and salt mat layer at different stages of Lake Magic. The statistical significance (ANOVA) is shown as asterisks.
